# Fodinibius alkaliphilus sp. nov., a moderately halophilic and alkaliphilic bacterium isolated from an inland saltern in central Portugal and reclassification of Aliifodinibius salipaludis as Fodinibius salipaludis sp. nov.

**DOI:** 10.1099/ijsem.0.006840

**Published:** 2025-07-09

**Authors:** Yang He, Marta Filipa Simões, Rafael R. de la Haba, Cátia Santos-Pereira, Joana Sousa, Joana S. Gomes, Sara C. Silvério, Lígia R. Rodrigues, André Antunes

**Affiliations:** 1 State Key Laboratory of Lunar and Planetary Sciences, Macau University of Science and Technology (MUST), Taipa, Macau SAR, PR China; 2 China National Space Administration (CNSA), Macau Center for Space Exploration and Science, Taipa, Macau SAR, PR China; 3 China-Portugal Belt and Road Joint Laboratory on Space & Sea Technology Advanced Research, PR China; 4 Department of Microbiology and Parasitology, Faculty of Pharmacy, University of Sevilla, Sevilla, Spain; 5 CEB – Centre of Biological Engineering, Universidade do Minho, Braga, Portugal; 6 LABBELS – Associate Laboratory, Braga/Guimarães, Portugal; 7 Institute of Science and Environment, University of Saint Joseph, Macau, Macau SAR, PR China

**Keywords:** bacteria, *Balneolota*, halophiles, inland saltern

## Abstract

A novel moderately halophilic and alkaliphilic Gram-stain-negative, strictly aerobic, bacterial strain (N2^T^) was isolated from an inland saltern in central Portugal. The taxonomic position of this isolate was determined based on polyphasic taxonomic and phylogenomic analysis. Phylogenetic analysis based on 16S rRNA gene sequences indicated that isolate N2^T^ belongs to the genus *Fodinibius*, showing the highest similarity to *Fodinibius halophilus* 2W32^T^ (98.14%). Phylogenomic analysis based on whole genomes, using the up-to-date bacterial core gene sets (92 genes), showed that strain N2^T^ formed a distinct monophyletic lineage within the genus *Fodinibius*. The cells of N2^T^ were motile rods that grew at temperatures between 30 and 40 °C (optimum at 35 °C), pH levels of 6.0–11.0 (optimum at pH 9.0) and salinities of 13–20 % (w/v) NaCl (optimum at 15% NaCl). Cells tested positive for oxidase and catalase activity. The predominant isoprenoid quinone was menaquinone-7 (MK-7), and the major fatty acids were iso-C_15:0_, anteiso-C_15:0_, C_16:1_* ω6c* and/or 10-methyl C_16:0_. The polar lipids included two aminolipids, two glycolipids and seven phospholipids. The DNA G+C content was 42.0 mol%. Based on phylogenetic, phylogenomic, genomic, phenotypic and chemotaxonomic data, we propose that strain N2^T^ (=KCTC 102228^T^=MCCC 1K08942^T^) represents a novel species within the genus *Fodinibius*, with the name *Fodinibius alkaliphilus* sp. nov. We also propose the reclassification of *Alifodinibius salipaludis* as *Fodinibius salipaludis* sp. nov.

## Data Availability

The authors confirm that all supporting data have been provided within the article. The GenBank/EMBL/DDBJ accession number for the 16S rRNA gene sequence of strain N2^T^ is PP669793. The draft genome sequence of strain N2^T^ has been deposited at DDBJ/ENA/GenBank under accession JBCFYD000000000.

## Introduction

The genus *Fodinibius*, belonging to the family *Balneolaceae* [1] in the phylum *Balneolota* [2], currently comprises seven recognized species with validly published names: *Fodinibius halophilus* [1], *Fodinibius roseus* [3], *Fodinibius salicampi* [4], *Fodinibius salinus* [5], *Fodinibius saliphilus* [6], *Fodinibius salsisoli* [7] and *Fodinibius sediminis* [3] (https://lpsn.dsmz.de/genus/fodinibius). Furthermore, and within this cluster, the species *Aliifodinibius salipaludis* [8] has been described, but its name has not yet been validly published. All these species were isolated from hypersaline environments, including salterns [1,4, 6], salt mines [3,5] and saline soils [7,8].

The Rio Maior Salterns are located in central Portugal, within the Serras de Aire e Candeeiros Natural Park. The brine, which is around seven times saltier than seawater, is pumped out of a well and is generated by the crossing of a local underground water stream with an extensive rock-salt deposit. Although first referenced in documents from the 12th century, it is believed that these sites have been linked with salt extraction since pre-historic times. They provide a unique setting, as they are the only inland salterns present in Portugal and the only fully functioning ones in all of Europe. Our study was part of a bioprospection campaign focusing on this site and resulted in the isolation of a bacterial strain (N2^T^) and the discovery of a putative new species within the genus *Fodinibius*. Here, we determine the taxonomic position of this new isolate based on phenotypic, chemotaxonomic and molecular properties.

## Sample collection and physicochemical characterization

During an investigation of the microbial diversity of hypersaline environments in central Portugal, samples were collected at Rio Maior Salterns, Portugal (39.36334 °N, 8.94502 °W), in July 2019. Sampling was performed using a telescopic sampler to collect water samples into sterile glass bottles. Temperature was determined on site with a thermometer. Samples were labelled, transported aseptically to the lab and stored at 4 °C until further analyses.

Collected samples were processed for physicochemical characterization. A Consort C3010 multiparameter analyser was used to measure salinity and pH. To obtain the concentration of sulphate (HAC-LCK 153), nitrate (HAC-LCK 339) and phosphate (HAC-LCS 349), Hach kits were used according to the manufacturer’s guidelines. The concentrations of Mg^2+^, Ca^2+^, Mn^2+^, Fe^2+^ and K^+^ were estimated by inductively coupled plasma optical emission spectrophotometry with the OPTIMA 8000 instrument (Perkin Elmer). Increasing concentration mixtures of Perkin Elmer Pure IV solution were prepared in 1% (v/v) nitric acid and analysed as calibration standards. The elements’ concentration was then determined using appropriate dilutions of the saltern water samples and resorting to the Syngistix^™^ software.

## Microbial isolation

During the diversity survey of the salterns of Rio Maior, strains assigned to a total of 12 genera were isolated, including *Aliifodinibius* and *Fodinibius* (*Balneolota*); *Haloarcula* (*Methanobacteriota*); *Aidingimonas*, *Chromohalobacter*, *Halomonas*, *Henriciella*, *Idiomarina*, *Marinobacter*, *Pseudomonas*, *Salicola* and *Spiribacter* (*Pseudomonadota*). Among these isolates, strain N2^T^ was retrieved from one of the water samples (Rio Maior B) collected at these Salterns (physicochemical data on this sample are available in Table 1). The original sample was diluted in marine broth (MB, BD Difco) with 12% (w/v) NaCl, and pH was adjusted to 7.0 using 1 M NaOH. The culture was incubated at 37 °C for 15 days. After repeated plate streaking (for three times) on marine agar 2216 (MA, BD Difco), an axenic culture was obtained and cryopreserved at −80 °C as a suspension in MB with 15% NaCl (w/v) and 20% (v/v) glycerol.

**Table 1.** Physicochemical characterization of the water sample collected at Rio Maior Saltern

Characteristic	Value
**Temperature (°C**)	26±1
**Salinity (g kg^−1^**)	291.0±6.5
**Salinity (%**)	29.1±0.7
**pH**	7.54±0.06
**SO_4_^2-^ (mg l^−1^**)	3750.0±21.2
**PO_4_^3-^-P (mg l^−1^**)	<0.15
**NO_3_^-^-N (mg l^−1^**)	0.6±0.1
**Mg^2+^ (mg l^−1^**)	197.4±1.1
**Ca^2+^ (mg l^−1^**)	988.0±14.5
**K^+^ (mg l^−1^**)	433.3±0.9
**Mn^2+^ (mg l^−1^**)	0.46±0.00
**Fe^2+^ (mg l^−1^**)	nd

nd – not detected.

## 16S rRNA gene phylogenetic analysis

Genomic DNA of strain N2^T^ was extracted and purified using the TSINGKE TSP701-50 Trelief^®^ Bacteria Genomic DNA Kit. The complete 16S rRNA gene was amplified by PCR using bacterial universal primers: 27F (5′-AGTTTGATCMTGGCTCAG-3′) and 1492R (5′-GGTTACCTTGTTACGACTT-3′) [9]. The obtained 16S rRNA gene sequence of strain N2^T^ is 1,399 nt in length.

**Fig. 1.**
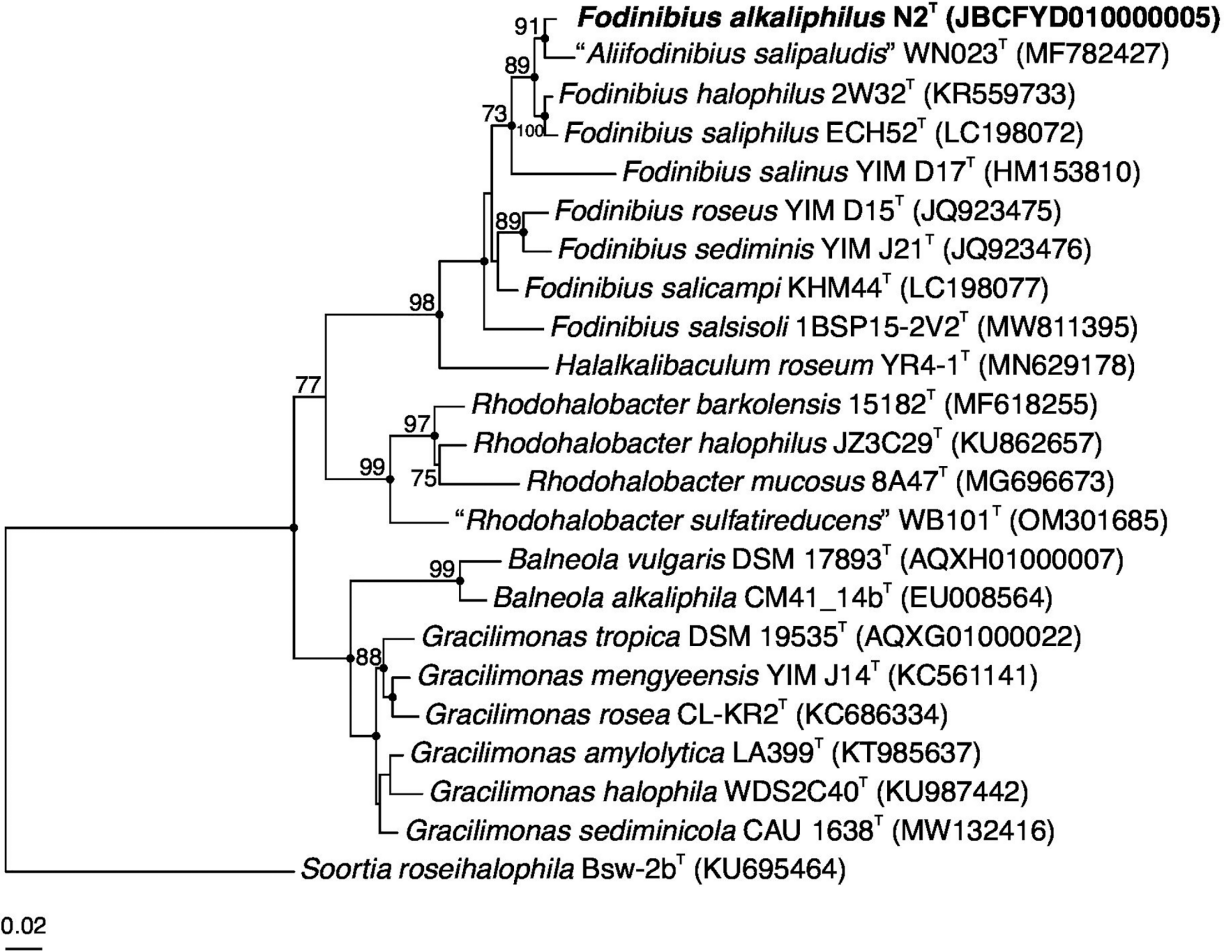
ML phylogenetic tree based on the 16S rRNA gene sequence comparison of strain N2^T^ and other related species within the family *Balneolaceae*. The species *Soortia roseihalophila* Bsw-2b^T^ was used as an outgroup. Sequence accession numbers are shown in parentheses. Bootstrap values higher than 70% are shown at branch points. Filled circles indicate branches that were recovered for the trees obtained using the ML, neighbour-joining and maximum-parsimony algorithms. Bar, 0.02 expected substitutions per nucleotide position.

Sequence analysis shows that the 16S rRNA gene of the strain N2^T^ has a similarity greater than 93% with the type strains from genus *Fodinibius*. The ARB software v.7.0 [10] was employed for sequence alignments and phylogenetic tree inference based on the sequence data of the 16S rRNA genes. Phylogenetic tree reconstructions were performed using maximum-likelihood (ML) [11], as implemented in IQ-TREE v.2.3.6 [12], and neighbour-joining [13] and maximum-parsimony [14] algorithms, as implemented in ARB software v.7.0. Branch support was assessed using 1,000 bootstrap [15] or ultrafast bootstrap [16] pseudo-replicates. The best-fit model selected for ML inference was TIM+F+R3, according to ModelFinder [17]. Relevant taxonomic sequences were sourced from the GenBank database (www.ncbi.nlm.nih.gov/genbank/, accessed on 7 November 2024). The ‘gitana’ script (https://github.com/cristinagalisteo/gitana) was used for formatting and visualization of the phylogenetic trees [18]. As illustrated in Fig. 1, the ML phylogenetic tree based on 16S rRNA gene sequences suggests that the strain N2^T^ clusters with members of the genus *Fodinibius*.

The similarity values among the sequences were determined using the EzTaxon server (https://eztaxon-e.ezbiocloud.net) [19]. Strain N2^T^ exhibits the highest sequence similarity of 98.14% with *F. halophilus* 2W32^T^ (KR559733). The strain’s 16S rRNA gene sequence shows similarities of 97.85% with *A. salipaludis* WN023^T^ (MF782427), 97.59% with *F. saliphilus* ECH52^T^ (LC198072), 95.49% with *F. salicampi* KHM44^T^ (LC198077), 94.92% with *F. sediminis* DSM 21194^T^ (JQ923476), 94.85% with *F. roseus* DSM 21986^T^ (JQ923475), 94.76% with *F. salsisoli* 1BSP15-2V2^T^ (MW811395) and 93.74% with *F. salinus* YIM D17^T^ (HM153810).

These results indicate that strain N2^T^ belongs to the genus *Fodinibius* and suggest that it likely represents a new species. Likewise, it highlights the need to rectify the current taxonomic status of *A. salipaludis*. From a nomenclatural perspective, this species has not been validly published under the International Code of Nomenclature of Prokaryotes, and its pending status should be resolved. Our results indicate that it should be reassigned to the genus *Fodinibius*, as the only reason for its current position is the fact that it had been described but not validly published when members of the genus *Aliifodinibius* were reclassified as *Fodinibius* [7].

## Phylogenomics and genome features

The extracted genomic DNA was quantified using 1% agarose gel electrophoresis, spectrophotometry (Nanodrop One spectrophotometer, Thermo-Fisher Scientific) and fluorometry (Qubit 3.0 fluorometer) with the Qubit dsDNA BR assay kit (Thermo-Fisher Scientific). Whole-genome sequencing of strain N2^T^ was performed using the Illumina HiSeq X platform by TSINGKE (Tsingke Biological Technology, Co., Ltd.), and the genome *de novo* assembly was carried out by using the software SPAdes v3.11.1 (http://cab.spbu.ru/software/spades/). The G+C content of the genome was determined according to the whole-genome sequence. The total genome of strain N2^T^ produced 991 Mbp of clean data, with an approximate sequencing depth of 297×. The genome size is 3.41 Mbp, with a chromosomal G+C content of 42.0 mol%. The statistical results of the gene prediction for strain N2^T^ are presented in Table S1, available in the online Supplementary Material (Supplementary Material 1). Additional gene analysis was done using RAST software [20], the National Center for Biotechnology Information (NCBI) Prokaryotic Genome Annotation Pipeline [21] and deposited in NCBI GenBank under accession JBCFYD000000000.

Publicly available genomes of strains belonging to the family *Balneolaceae* were downloaded from NCBI GenBank to refine the comparative genome analysis. The average nucleotide identity (ANI) values between the genome of strain N2^T^ and those of eight closest related strains were calculated using EzBioCloud [22]. The results showed that the ANI values ranged from 71.22 to 76.77% (Table S2; Supplementary Material 1).

**Fig. 2.**
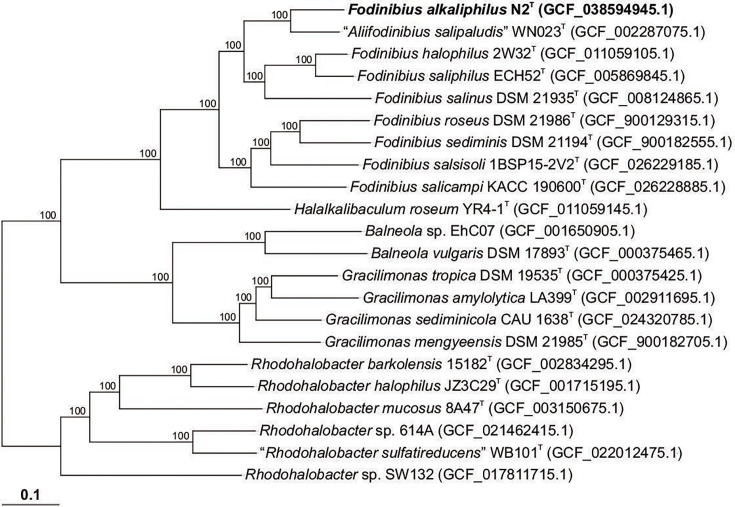
ML phylogenomic tree based on the comparison of 990 core orthologous proteins showing the relationships between strain N2^T^ and members of the family *Balneolaceae*. Assembly accession numbers are shown in parentheses. Ultrafast bootstrap values (%) are shown at branch points. Bar, 0.1 substitutions per amino acid position.

Strain N2^T^ and 21 related strains in the family *Balneolaceae* were used to construct a phylogenomic tree based on single-copy core orthologous protein clusters. Briefly, the single-copy core proteins were identified using all-vs.-all blastp v.2.10.1+ comparisons among the translated coding sequences of the annotated genomes under study, followed by clustering with the Markov Cluster Algorithm implemented in the Enveomics toolbox [23]. The single-copy core protein sequences were individually aligned with muscle v.5.1 [24] and then concatenated. An ML phylogenomic tree was generated using IQ-TREE v.2.3.6 [12], employing partitioned analysis for multi-protein alignments. ModelFinder [17], as implemented in IQ-TREE v.2.3.6, was employed to choose the right partition scheme. Tree branch support was inferred using the ultrafast bootstrap approximation [16]. The resulting phylogeny was visualized using the iTOL website [25]. The phylogenomic tree (Fig. 2) revealed that strain N2^T^ clustered with *A. salipaludis* WN023^T^, with a bootstrap value of 100, and the type strains of the species of the genus *Fodinibius*. These results are in agreement with the ones obtained from the 16S rRNA gene phylogenetic analysis.

The genome comprised a total of 3,007 genes, including 2,956 protein-coding genes, 39 tRNA genes and 3 rRNA genes (one 5S, one 16S and one 23S). The annotations Clusters of Orthologous Genes (COG), Gene Ontology (GO), Kyoto Encyclopedia of Genes and Genomes (KEGG) and CAZymes were predicted using blast programs (https://blast.ncbi.nlm.nih.gov/Blast.cgi). According to the COG database annotation results (Fig. S1; Supplementary Material 1), the largest categories were those related to amino acid transport and metabolism (10.25%), followed by translation, ribosomal structure and biogenesis (7.61%), cell wall/membrane/envelope biogenesis (7.28%), energy production and conversion (6.78%) and post-translational modification, protein turnover and chaperones (5.94%). COG genes of unknown function accounted for the highest proportion (15.91%), suggesting that genes present in N2^T^ contributing to other functions deserve further exploration. According to the GO categories results (Fig. S2; Supplementary Material 1), genes were classified into three GO functional categories: describing the cellular component, biological process and molecular function. A total of 73.53% of genes are related to catalytic activity, 65.43% to cellular processes and 59.85% to metabolic processes. KEGG analysis showed that functional and metabolic pathways were divided into six categories. Of these, most genes were enriched in metabolism (76.80%), followed by genetic information processing (10.77%), cellular processes (4.97%), environmental information processing (4.97%), human diseases (1.85%) and organismal systems (0.64%) (Fig. S3; Supplementary Material 1). This result indicates that a large number of annotated genes in N2^T^ are metabolically functional and have metabolic pathways essential for primary metabolism. According to CAZymes categories analysis, many carbohydrate-active genes were annotated in the genome of strain N2^T^, which can encode carbohydrate-active enzymes, including 67 glycoside hydrolases, 92 glycosyltransferases, 21 polysaccharide lyases, 54 carbohydrate esterases, 39 auxiliary activities and 20 carbohydrate-binding modules (Fig. S4; Supplementary Material 1).

Given the physicochemical characteristics of the original sample, we were interested in analysing the genomic data of N2^T^ to identify some genes associated with resistance to high-salinity and alkaline conditions. As a result of annotation and survey, genes encoding MrpABCDEH, NhaA and NptA were detected (Fig. S5; Supplementary Material 1). Mrp complexes and NhaA are bacterial Na^+^/H^+^ antiporters associated with resistance to high-salinity and alkaline pH conditions [26,31]. NptA is a sodium-dependent phosphate transporter that may function as an adaptation to changes in salinity or pH in the environment [32,33]. The presence of these genes aligns well with the conditions observed in the original environment from which this strain was isolated.

## Morphology, physiology and biochemical analysis

Gram staining was carried out using a Gram stain kit (Thermo-Fisher Scientific). Motility was examined using the hanging-drop method [34]. Growth conditions of strain N2^T^ were tested as follows. For growth temperature tests, the strain was inoculated on MA supplemented with 12% (w/v) NaCl at different temperatures (15, 25, 30, 35, 40 and 45 °C) for 10 days, until growth was detected by visible colonies (growth development was checked and recorded every 12 h). The growth salinity range and optimum salinity were tested using a medium consisting of (g l^−1^): tryptone (CM0129 Oxoid), 10; yeast extract (IB499160 IBI Scientific), 5; agar (Acros Organics), 15, with varying concentrations of NaCl (0, 3, 6, 10, 15, 18, 20, 23, 25 and 28%, w/v), at 37 °C, and visual recording of growth every 12 h. The pH range for growth was determined in MB supplemented with 12% (w/v) NaCl, at pH 4.0–12.0, at intervals of 1 pH unit with citrate/phosphate (pH 4.0–7.0), Tris/HCl (pH 8.0–9.0) and Na_2_CO_3_/NaHCO_3_ (9.0–12.0). Furthermore, OD at 600 nm (OD_600_) of the culture was measured after 72 h of incubation at 37 °C. Anaerobic growth was determined by incubation of inoculated MA supplemented with 12% (w/v) NaCl, using the AnaeroPack^™^ system (Mitsubishi Gas Chemical Co., Tokyo, Japan), for 5 days at 37 °C.

Colonies grown on MA plates supplemented with 12% (w/v) NaCl were pink. Cells observed under an optical microscope (Panthera C-coded, Motic) exhibited a Gram-negative profile. N2^T^ showed distinct growth conditions, as detailed in Table 2. Strain N2^T^ grew within a temperature range of 30–40 °C, with an optimal growth temperature of 35 °C. The minimum growth temperature (30 °C) of the strain is higher than that of other reference strains. Strain N2^T^ grew in salinities ranging from 13 to 20% NaCl (w/v), with the optimal salinity for growth being 15%, which is the highest among the reference strains. The strain grew in pH levels from 6 to 11, with an optimum pH of 9, making it more suitable for alkaline growth compared to other reference strains.

**Table 2.** Characteristics that differentiate strain N2^T^ and type strains of the most closely related species 1, N2^T^; 2, *F. saliphilus* ECH52^T^; 3, *F. halophilus* KCTC 42497^T^; 4, *F. roseus* KCTC 23442^T^; 5, *F. sediminis* DSM 21194^T^; 6, *F. salicampi* KHM44^T^; 7, *F. salinus* YIM D17^T^; 8, *F. salsisoli* 1BSP15-2V2^T^; 9, *A. salipaludis* WN023^T^. All strains are Gram-negative and moderately halophilic rods that are positive for the catalase reaction.

Characteristic	1	2^*a*^	3^*a*^	4^*a*^	5^*a*^	6^*a*^	7^*b*^	8^*c*^	9^*d*^
Habitat	Inland saltern	Saltern	Saltern	Salt mine	Salt mine	Saltern	Salt mine	Soil	Soil
Colony colour	Salmon pink	Red	Reddish	Rose red	Salmon pink	Salmon pink	Pink	Orange-red	Brown
Motility	Motile	Non-motile	Non-motile	Non-motile	Non-motile	Non-motile	Non-motile	Non-motile	Non-motile
Temperature range for growth (°C)	30–40	28–45	20–45	20–42	25–45	20–45	25–45	14–43	15–45
Optimum	35	37	33–37	28	28	37	37	37	nd
pH range for growth	6.0–11.0	6.0–10.0	7.0–8.5	6.5–8.0	6.5–8.5	6.0–9.0	6–9	5–8	6.5–11.0
Optimum	9.0	8.0	7.5–8.0	7.0	7.0	8.0	7.5–8.0	6	nd
NaCl range for growth (%, w/v)	13–20	5–25	2–18	4–20	4–16	3–25	4–23	3–20	5–25
Optimum	15	10	8	8	8	10	10–15	9	nd
Enzyme activities (API ZYM):									
Esterase (C4)	+	+	+	+	−	+	−	nd	+
*α*-Chymotrypsin	+	+	+	−	+	−	−	nd	−
*N*-Acetyl-*β*-glucosaminidase	+	+	+	−	+	+	+	nd	−
*β*-Galactosidase	W/+	−	−	+	+	+	−	nd	−
Trypsin	+	−	−	−	+	−	−	nd	−
Polar lipids	DPG, PC, PE, AL, GL, L	DPG, PC, PE, GL, L	DPG, PC, PE, GL, L, AL^*e*^	DPG, PC, PE, GL, L, PL^*b*^	DPG, PC, PE, GL, L, PL^*b*^	DPG, PC, PE, GL, L	DPG, PE, PC, PG, GL, AL, PL, L	nd	L, GL, AL, PL, PC, PE, DPG
DNA G+C content (mol%)	42.0*	40.8	47.5^*e*^	49.0^*b*^	48.4^*b*^	48.5	43	44.5	42.2

Data from: *a*, Cho and Whang [6]; *b*, Wang *et al*. [5]; *c*, Galisteo *et al*. [7]; *d*, Zhao *et al*. [8]; *e*, Xia *et al*. [1].

*Whole-genome sequence data.

+, Positive; −, Negative; W, Weak positive; nd, No data.

AL, aminolipid; DPG, diphosphatidylglycerol; GL, glycolipid; L, unknown polar lipid; PC, phosphatidylcholine; PE, phosphatidylethanolamine; PG, Phosphatidylglycerol; PL, Unknown phospholipid.

Catalase activity was determined by dropping a 3.0% H_2_O_2_ solution onto collected, freshly grown biomass. Oxidase activity was evaluated using the oxidase test dipstick (Hangzhou Microbial Reagent Co., Ltd.). Other physiological and biochemical features were determined using the services available at the Marine Culture Collection of China (MCCC) and assessed using API 20NE, API 50CH and API ZYM strips (bioMérieux) according to the manufacturer’s instructions, with the single modification of adjusting the NaCl concentration to 15.0% (w/v) in all tests. Strain N2^T^ exhibited positive catalase and oxidase activities. Enzyme activity was observed for alkaline phosphatase, esterase (C4), esterase lipase (C8), lipase (C14), leucine arylamidase, valine arylamidase, cystine arylamidase, trypsin, *α*-chymotrypsin, acid phosphatase, naphthol-AS-BI-phosphohydrolase and *N*-acetyl-*β*-glucosaminidase, with weak activity for *α*-galactosidase and *β*-galactosidase (API ZYM). Positive results were also obtained for aesculin hydrolysis and *β*-galactosidase activity (API 20NE). Does not ferment glycerol, erythritol, d-arabinose, l-arabinose, d-ribose, d-xylose, l-xylose, d-adonitol, methyl-*β*-d-xylopyranoside, d-galactose, d-glucose, d-fructose, d-mannose, l-sorbose, l-rhamnose, dulcitol, inositol, d-mannitol, d-sorbitol, methyl-*α*-d-mannopyranoside, methyl-*α*-d-glucopyranoside, *N*-acetylglucosamine, amygdalin, arbutin, aesculin, salicin, d-cellobiose, d-maltose, d-lactose, d-melibiose, d-sucrose, d-trehalose, inulin, d-melezitose, d-raffinose, starch, glycogen, xylitol, *β*-gentiobiose, d-turanose, d-lyxose, d-tagatose, d-fucose, l-fucose, d-arabitol, l-arabitol, gluconate, 2-keto-gluconate and 5-keto-gluconate (API 50CH). Additional results from biochemical and physiological characteristics of strain N2^T^ and related species are summarized in Table 2 and in the species description.

Susceptibility to antibiotics was tested on MA plates supplemented with 12% (w/v) NaCl using antibiotic discs (Hangzhou Microbial Reagent Co., Ltd.). Cells of strain N2^T^ were susceptible to teicoplanin (30 µg), ampicillin (30 µg), zeomicin (2 µg), nalidixic acid (30 µg), penicillin G (10 µg), vancomycin (30 µg), erythromycin (15 µg), amoxicillin (20 µg), spiramycin (30 µg), rifampicin (5 µg), tetracycline (30 µg), roxithromycin (15 µg), phosphomycin (200 µg) and chloramphenicol (30 µg). However, they were resistant to amikacin (30 µg), kanamycin (30 µg), gentamicin (10 µg), streptomycin (10 µg), neomycin (30 µg), polymyxin (300 µg), mycopeptides (0.04 µg) and amphotericin (30 µg).

## Chemotaxonomic characterization

Fatty acid and lipid profiles, as well as respiratory quinones, were determined after cultivation for 72 h at 37 °C using the services available at MCCC. For fatty acid characterization, cells of strain N2^T^ were harvested after cultivation on MA supplemented with 12% (w/v) NaCl. The cellular fatty acids were extracted according to the Sherlock Microbial Identification System (MIDI, version 6.1) [35]. The fatty acids were analysed by GC (Agilent Technologies 6850) and identified using the TSBA6.0 database of the MIDI [35].

To determine polar lipids, cells of strain N2^T^ were harvested after cultivation in MB medium supplemented with 12% (w/v) NaCl and then freeze-dried. Polar lipids were extracted using a chloroform/methanol system and analysed via two-dimensional TLC, as described previously [36]. Silica gel 60 F254 aluminium-backed thin-layer plates (Merck) were used in TLC analysis. The plate dotted with the sample was subjected to two-dimensional development, with the first solvent being chloroform-methanol-water (65:25:4, by vol.) and the second being chloroform-methanol-acetic acid-water (80:12:15:4, by vol.). Total lipid material was detected using molybdatophosphoric acid, and specific functional groups were detected using spray reagents specific for defined functional groups [36].

Extracted lipids were identified by spraying TLC plates with appropriate detection reagents. Total lipids were detected by spraying a plate with 10% ethanolic molybdophosphoric acid, followed by charring at 180 °C for 15 min (Fig. S6-A; Supplementary Material 1). A second plate was sprayed with 1-naphthol-sulphuric acid, revealing the presence of glycolipids as brown spots, after heating at 110 °C for 15 min (Fig. S6-B; Supplementary Material 1) [37]. Amino lipids were detected by spraying with ninhydrin (0.4% in water-saturated butanol), heated at 110 °C for 15 min and pink spots marked lightly with pencil (Fig. S6-C; Supplementary Material 1), while the same plate was later sprayed with the lipid phosphate reagent of Dittmer and Lester (molybdenum blue reagent) to reveal the presence of phospholipids as blue spots (Fig. S6-D; Supplementary Material 1).

For quinone characterization, cells of strain N2^T^ were collected after cultivation in MB supplemented with 12% (w/v) NaCl. Respiratory quinones were analysed by HPLC [38].

The major fatty acids determined for strain N2^T^ were iso-C_15:0_ (46.62%), anteiso-C_15:0_ (14.19%), C_16:1_* ω7c* and/or C_16:1_* ω6c* (7.47%) and iso-C_17:1_* ω9c* and/or 10-methyl C_16:0_ (7.32%) (full profile available in Table 3). Polar lipids comprised two aminolipids (AL1 and AL2), two glycolipids (GL1 and GL2) and seven phospholipids (DPG, PE, PC and L1–L4) (Fig. S6; Supplementary Material 1). The extracted quinone was identified as menaquinone-7 (MK-7). These results are in agreement with those observed for other species of the genus *Fodinibius* [1,3,8].

**Table 3.** Cellular fatty acid compositions (%) of strain N2^T^ and related type strains 1, N2^T^; 2, *F. saliphilus* ECH52^T^; 3, *F. halophilus* KCTC 42497^T^; 4, *F. roseus* KCTC 23442^T^; 5, *F. sediminis* DSM 21194^T^; 6, *F. salicampi* KHM44^T^; 7, *F. salinus* YIM D17^T^; 8, *F. salsisoli* 1BSP15-2V2^T^; 9, *A. salipaludis* WN023^T^. Major components (>5.0 %) are highlighted in bold; –, not detected; tr, trace amount (<1%).

Fatty acid	1	2^*a*^	3^*a*^	4^*a*^	5^*a*^	6^*a*^	7^*b*^	8^*c*^	9^*d*^
**C_14:0_**	2.0	–	–	–	–	–	tr	–	tr
**C_15:0_**	–	2.2	1.3	–	–	3.6		–	
**C_16:0_**	4.9	1.7	2.0	2.6	2.1	2.8	3.7	2.2	2.8
**C_18:0_**	tr	–	–	–	–	–	1.4	–	tr
**iso-C_15:0_**	**46.6**	**19.2**	**28.4**	**19.2**	**16.0**	**29.5**	**23.6**	**36.4**	**33.3**
**iso-C_15:1_ F**	tr	2.1	1.9	1.1	1.2	tr	2.4	tr	–
**iso-C_17:1_* ω9c***	–	**26.5**	**30.6**	**10.2**	**15.5**	**10.3**	–	–	**16.7**
**iso-C_16:0_**	1.3	**9.2**	4.2	**9.0**	**7.9**	3.8	3.8	1.7	–
**iso-C_17:0_**	2.0	1.2	1.7	1.6	2.3	2.0	tr	tr	–
**iso-C_17:0_ 3-OH**	2.5	tr	tr	1.5	–	1.6	tr	1.3	–
**anteiso-C_15:0_**	**14.2**	1.9	1.2	**21.0**	**18.4**	**7.4**	**8.1**	**8.2**	**5.8**
**anteiso-C_17:0_**	2.0	tr	tr	3.9	**6.8**	1.3	tr	tr	–
**anteiso-C_17:1_* ω9c***	tr	tr	tr	2.8	3.4	tr	1.4	–	2.4
**C_15:1_* ω6c***	tr	1.9	tr	1.9	tr	2.8	tr	2.8	–
**C_16:1_*ω5c***	1.5	2.7	3.2	2.3	2.9	2.3	1.5	**5.1**	2.5
**C_17:1_* ω6c***	tr	2.7	1.4	1.8	tr	1.5	tr	1.1	–
**C_17:1_* ω8c***	tr	2.3	1.2	1.7	tr	2.2	tr	1.4	–
**C_18:1_* ω9c***	tr	–	–	tr	tr	–	4.5	tr	–
**10-methyl C_18:0_**	–	–	–	–	–	–	3.1	–	–
**C_16:1_* ω7c*/iso-C_15:0_ 2-OH/C_16:1_* ω6c***	**7.5**	**16.8**	**15.2**	**14.3**	**12.9**	**22.7**	**13.8**	**24.7**	**20.5**
**iso-C_17:1_ and/or C_17:1_**	–	–	–	–	–	–	–	–	–
**iso-C_17:0_ 3-OH**	2.5	tr	tr	1.5	–	1.6			
**iso-C_17:1_* ω9c*/10-methyl C_16:0_**	**7.3**	–	–	–	–	–	**24**	**10.7**	**16.7**

Data from: *a*, Cho and Whang [6]; *b*, Wang *et al*. [5]; *c*, Galisteo *et al*. [7]; *d*, Zhao *et al*. [8].

The strain N2^T^ exhibits some typical characteristics of the family *Balneolaceae*, such as DNA G+C content and pigmentation. The phylogenetic tree based on the 16S rRNA gene and genomic analysis suggests that strain N2^T^ can be classified within the genus *Fodinibius*. In terms of phenotypic characteristics, strain N2^T^ shows some distinctive features when compared with the other type strains, namely a higher optimal salinity for growth and a preference for alkaline conditions.

In summary, phenotypic features, phylogenetic analysis and chemotaxonomic characterization indicate that strain N2^T^ can be distinguished from other species of the genus *Fodinibius*. Therefore, we propose that it represents a novel species, with the name *Fodinibius alkaliphilus* sp. nov. Furthermore, we propose that *A. salipaludis* should be reassigned to the genus *Fodinibius* as *Fodinibius salipaludis* sp. nov.

## Description of *Fodinibius alkaliphilus* sp. nov.

*Fodinibius alkaliphilus* (al.ka.li’phi.lus. N.L. n. *alkali*, from Arabic *al-qaliy* the soda ash; Gr. adj. *philos*, loving; N.L. masc. adj. *alkaliphilus*, loving alkaline conditions).

Cells are Gram-stain-negative, motile and rod-shaped (0.3–0.4×0.05 µm). Growth is not observed under anaerobic conditions. Colonies are salmon-coloured, round and transparent when grown on MA supplemented with 12% (w/v) NaCl at 37 °C for 3 days. Grows between 13 and 20% (w/v) NaCl (optimal at 15%), pH 6.0–11.0 (optimal at pH 9.0) and 30–40 °C (optimal at 35 °C). Catalase and oxidase positive. Negative for nitrate and nitrite reduction. Enzyme activity was observed for alkaline phosphatase, esterase (C4), esterase lipase (C8), lipase (C14), leucine arylamidase, valine arylamidase, cystine arylamidase, trypsin, *α*-chymotrypsin, acid phosphatase, naphthol-AS-BI-phosphohydrolase, *β*-galactosidase and *N*-acetyl-*β*-glucosaminidase, but not for *β*-glucuronidase, *α*-glucosidase, *α*-mannosidase, *β*-fucosidase, tryptophan deaminase, arginine dihydrolase, urease and gelatin hydrolysis. Weakly positive for *α*-galactosidase and variable result for *β*-glucosidase. Assimilates d-glucose, l-arabinose, d-mannose, d-mannitol, *N*-acetyl-glucosamine, d-maltose, potassium gluconate, capric acid, adipic acid, malate, trisodium citrate and phenylacetic acid. The major fatty acids (>5%) are iso-C_15:0_, anteiso-C_15:0_, C_16:1_* ω6c* and/or 10-methyl C_16:0_. The polar lipids profile consists of diphosphatidylglycerol, phosphatidylcholine, phosphatidylethanolamine, four unidentified lipids, two unidentified aminolipids and two unidentified glycolipids. The main respiratory quinone is menaquinone-7 (MK-7).

The type strain N2^T^ (KCTC 102228^T^=MCCC 1K08942^T^) was isolated from a water sample collected from an inland saltern in Rio Maior, central Portugal. The genome of the type strain has an approximate size of 3.41 Mb and a G+C content of 42.0 mol%. The accession number for its 16S rRNA gene sequence is PP669793 and that of the genome sequence is JBCFYD000000000.

## Description of *Fodinibius salipaludis* sp. nov.

*Fodinibius salipaludis* (sa.li.pa.lu’dis. L. masc. n. *sal*, salt; L. fem. n. *palus*, swamp, marsh; N.L. gen. n. *salipaludis* of a salt marsh).

The description is as given in the original proposal of *A. salipaludis* by Zhao *et al.* [8].

The type strain is WN023^T^ (=KCTC 52855^T^=ACCC 19978^T^). Isolated from natural saline-alkali wetland soil (Tianjin, China). The genome of the type strain has an approximate size of 3.58 Mb and has a G+C content of 42.2 mol%. The accession number for its 16S rRNA gene sequence is MF782427 and that of the genome sequence is NSKE00000000.

## Supplementary material

10.1099/ijsem.0.006840 Supplementary Material 1.

## Abbreviations

ANI: average nucleotide identity
BLAST: Basic Local Alignment Search Tool
COG: Clusters of Orthologous Genes
DDBJ: DNA Databank of Japan
EMBL: European Molecular Biology Lab
ENA: European Nucleotide Archive
GO: Gene Ontology
KCTC: Korean Collection for Type Cultures
KEGG: Kyoto Encyclopedia of Genes and Genomes
MA: marine agar 2216
MB: marine broth
MCCC: Marine Culture Collection of China
MCCC: Marine Culture Collection of China
ML: maximum-likelihood
NCBI: National Center for Biotechnology Information
T: type strain
