# Supplementary material for: Fodinibius alkaliphilus sp. nov., a moderately halophilic and alkaliphilic bacterium isolated from an inland saltern in central Portugal and reclassification of Aliifodinibius salipaludis as Fodinibius salipaludis sp. nov
Source: Int J Syst Evol Microbiol. 2025 Jul 9;75(7):006840. doi: 10.1099/ijsem.0.006840 (PMC12260241; doi:10.1099/ijsem.0.006840)
Supplement: Supplementary Material 1. [file ijsem-75-06840-s001.pdf]

Supplementary Material

Supplementary Figures

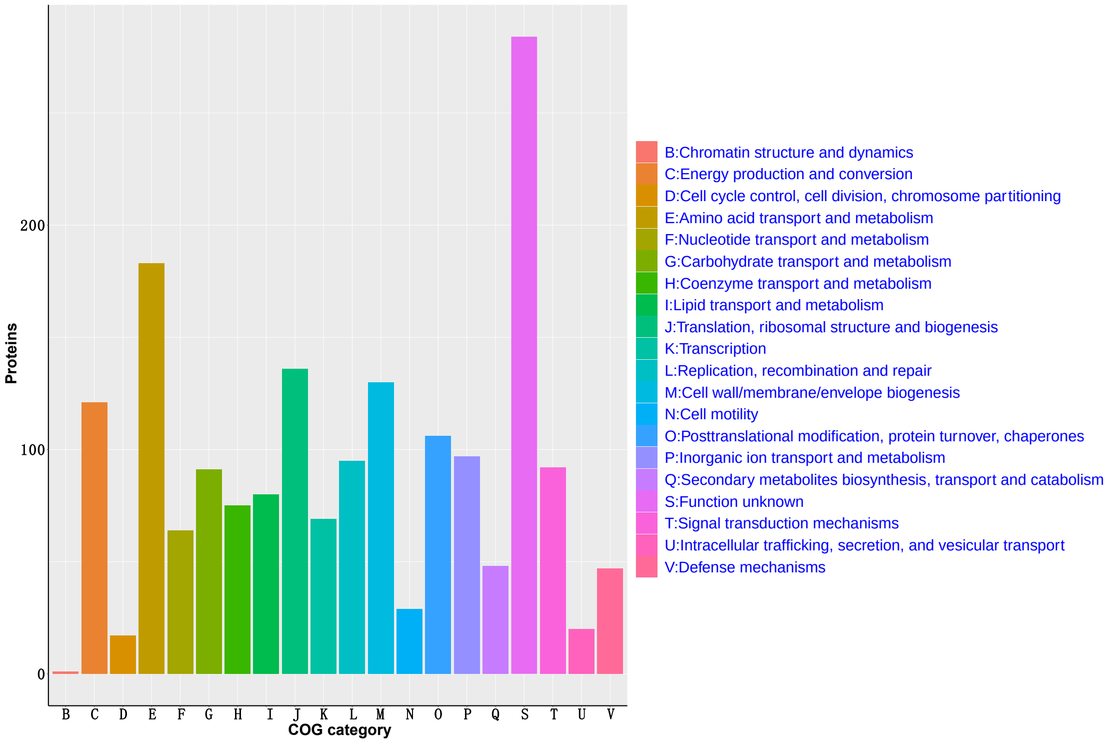

Figure S1 Annotation results of the Clusters of Orthologous Genes (COG) databases of the genome of strain N2^{T}.

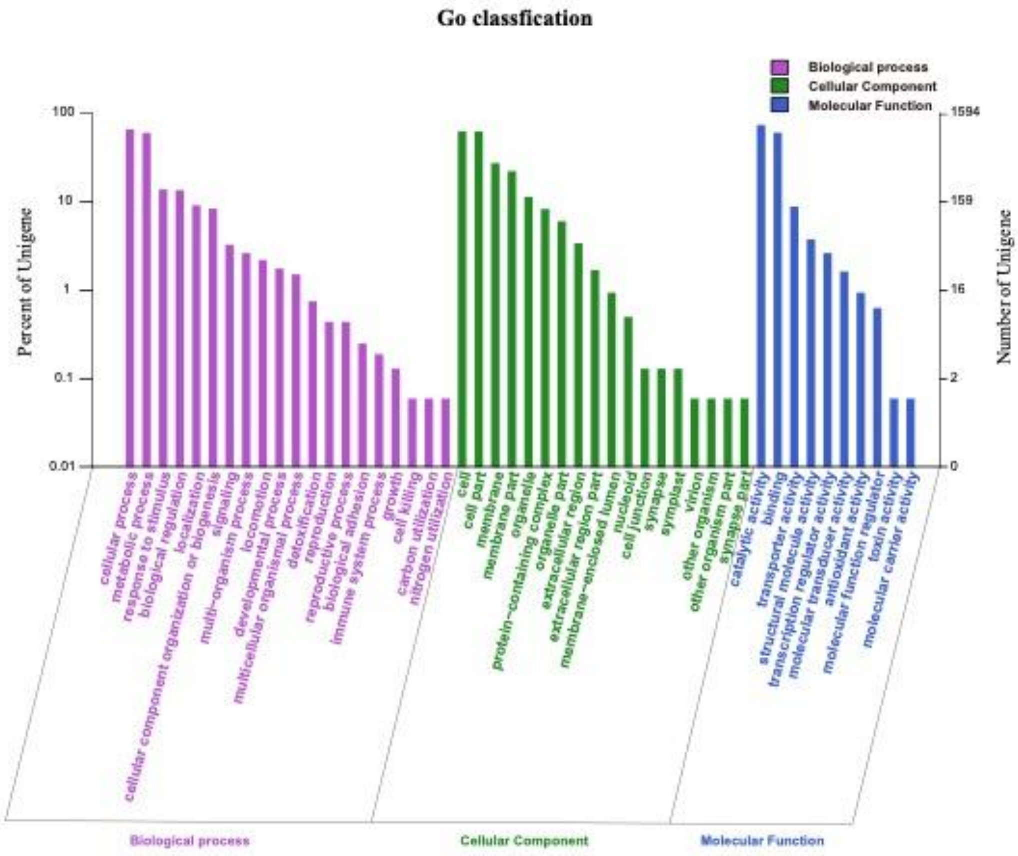

Figure S2 Annotation results of the Gene Ontology (GO) databases of the genome of strain N2^T.

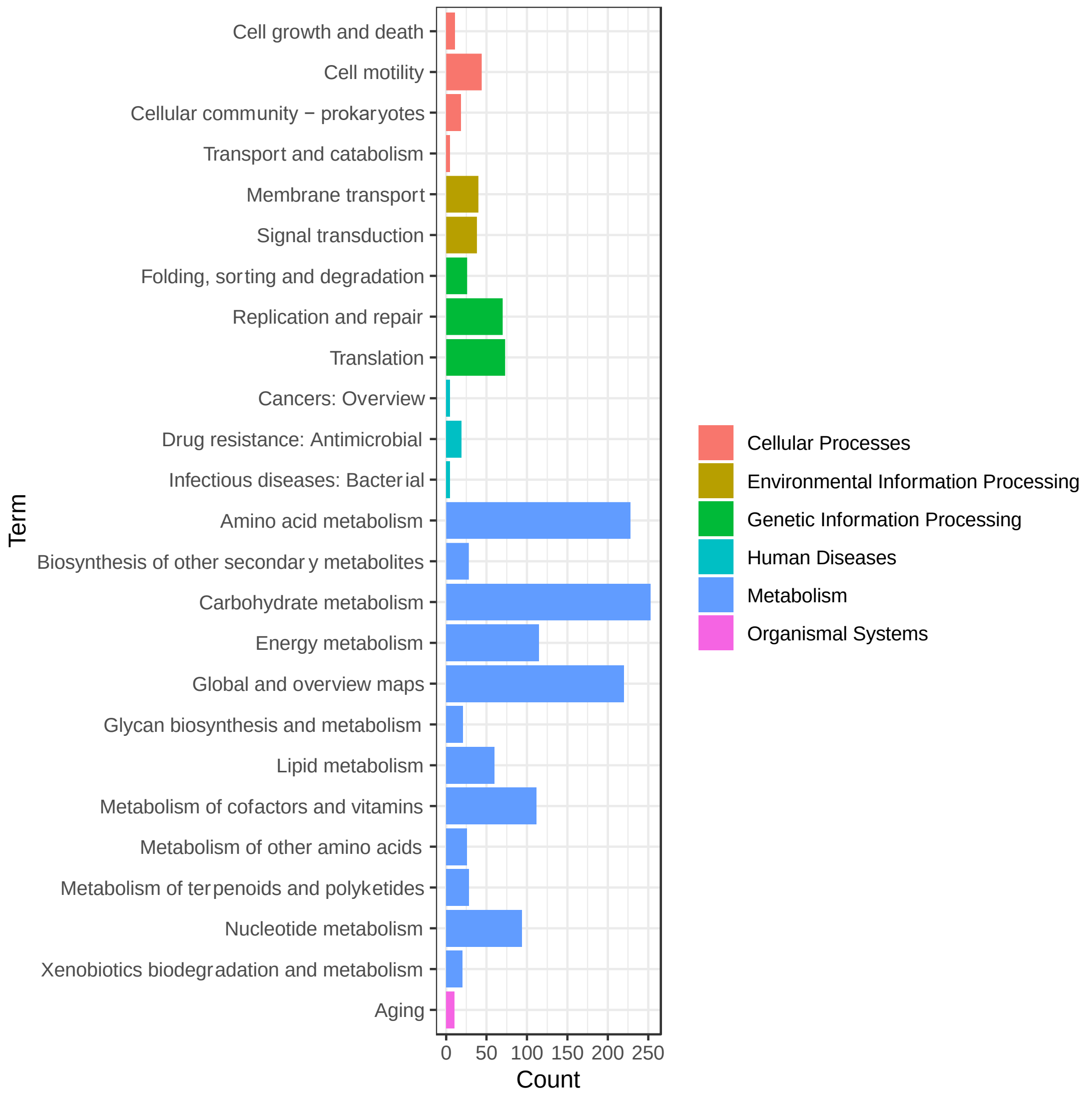

Figure S3 Annotation results of the Kyoto Encyclopedia of Genes and Genomes (KEGG) databases of the genome of strain N2^{T}.

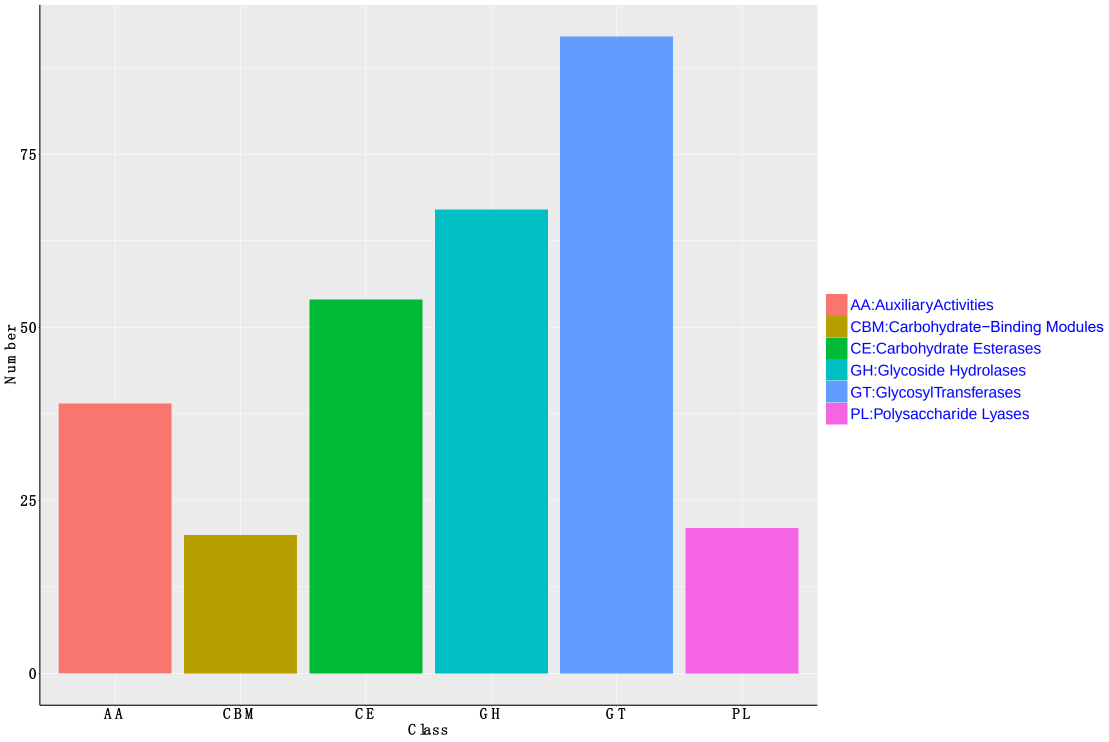

Figure S4 Summary of CAZymes categories identified in the genome of stain $N2^T$.

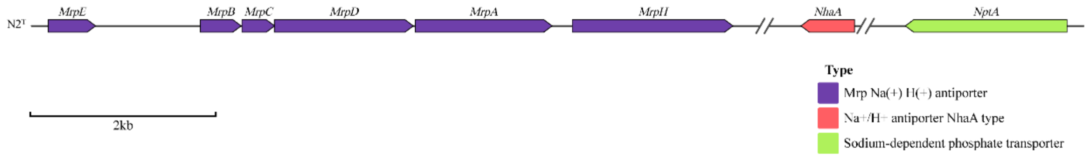

Figure S5. Composition of the gene cluster for resistance to high-salt and alkaline conditions for strain N2^T.

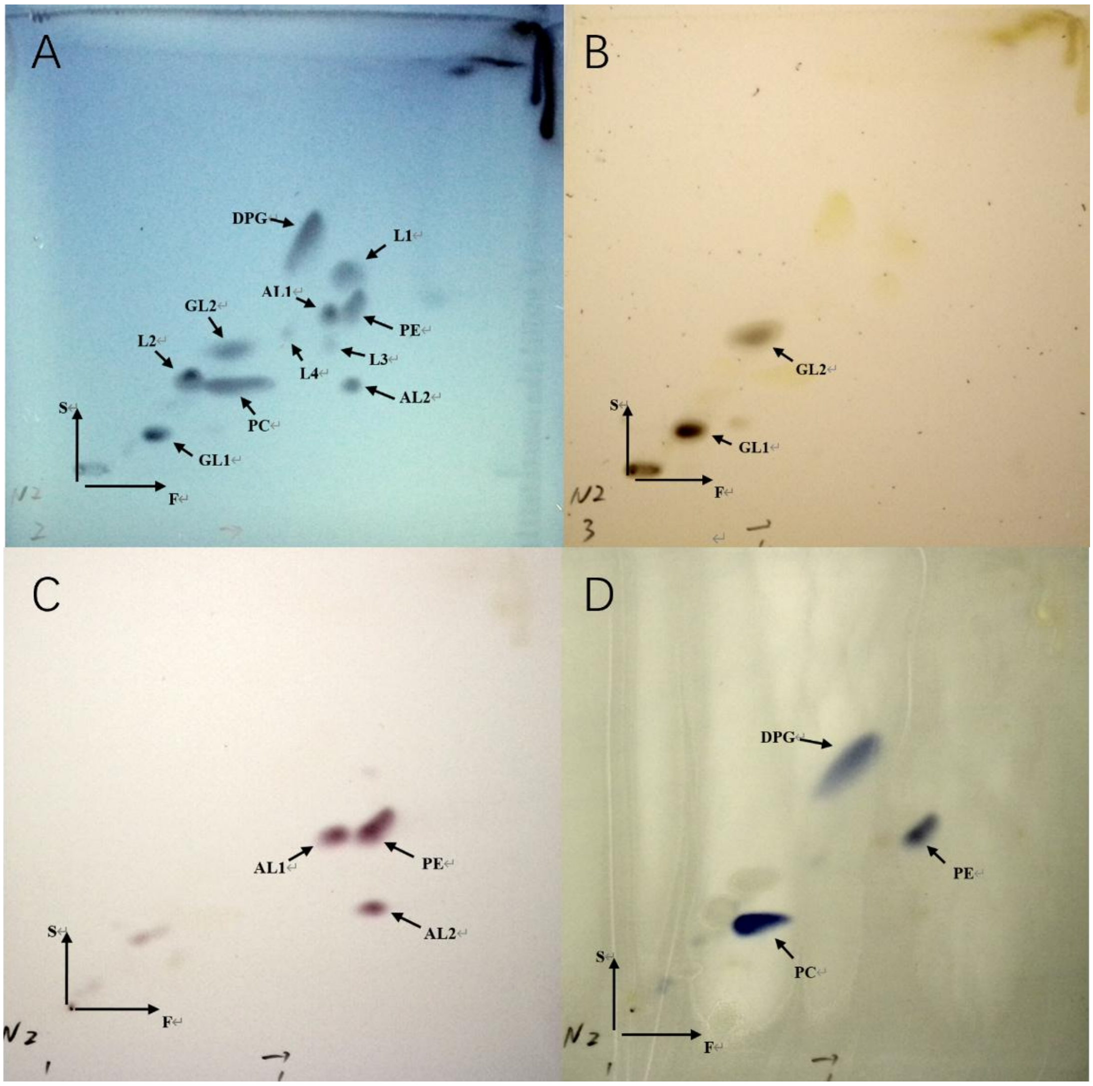

Figure S6. Polar lipids profile of strain N2^{T}. (**A**) total lipids, (**B**) glycolipids, (**C**) aminolipids, and (**D**) phospholipids. AL, aminolipid; DPG, diphosphatidylglycerol; GL, glycolipid; L, unknown polar lipid; PC, phosphatidylcholine; PE, phosphatidylethanolamine.

Supplementary Tables

Table S1. Gene prediction results of strain N2^T

Category	N2^T
Protein coding genes	2956
tRNA genes	39
rRNA genes	3
Total gene length	3075337
Average gene length	1014
G+C content in gene region	42.0%
Gene density(genes/Mb)	910
Gene/Genome (%)	90%
Intergenetic region length	334216

Table S2. Average nucleotide identity (ANI) between strain N2^T and reference strains

**Reference strains**	**ANI**
Fodinibius saliphilus ECH52^T	71.66%
Fodinibius halophilus 2W32^T	72.21%
Fodinibius roseus KCTC 23442^T	71.27%
Fodinibius sediminis DSM 21194^T	71.32%
Fodinibius salicampi KHM44^T	71.22%
Fodinibius salinus YIM D17^T	72.24%
"*Aliifodinibius salipaludis*" WN023^T	76.77%
Fodinibius salsisoli 1BSP15-2V2^T	71.79%
